# Pristine ices in a planet-forming disk revealed by heavy water

**DOI:** 10.1038/s41550-025-02663-y

**Published:** 2025-10-15

**Authors:** Margot Leemker, John J. Tobin, Stefano Facchini, Pietro Curone, Alice S. Booth, Kenji Furuya, Merel L. R. van ’t Hoff

**Affiliations:** 1 Dipartimento di Fisica, Università degli Studi di Milano, Milan, Italy; 2 National Radio Astronomy Observatory, Charlottesville, VA USA; 3 Departamento de Astronomía, Universidad de Chile, Santiago, Chile; 4 Center for Astrophysics, Harvard & Smithsonian, Cambridge, MA USA; 5 Department of Astronomy, Graduate School of Science, University of Tokyo, Tokyo, Japan; 6 RIKEN Pioneering Research Institute, Saitama, Japan; 7 Department of Physics and Astronomy, Purdue University, West Lafayette, IN USA

**Keywords:** Astrophysical disks, Astronomy and astrophysics

## Abstract

Water is essential to our understanding of the planet-formation process and habitability on Earth. Although trace amounts of water are seen across all phases of star and planet formation, the bulk of the water reservoir often goes undetected, hiding crucial parts of its journey from giant molecular clouds to planets. This raises the question of whether water molecules in comets and (exo-)planets is largely inherited from the interstellar medium or whether the water molecules are destroyed and then reformed in the disk. Water isotopologue ratios involving doubly deuterated water (D_2_O) are a sensitive tracer to answer this question. Here we present strong evidence of inheritance through an enhancement of D_2_O in the outbursting V883 Ori disk. The high D_2_O/H_2_O ratio of (3.2 ± 1.2) × 10^−5^ is consistent with values seen in protostellar envelopes and a comet and is 2 orders of magnitude higher than expected if water is reprocessed. The high deuteration of the heaviest isotopologues D_2_O/HDO = (2.3 ± 1.0) × HDO/H_2_O further establishes the inheritance of water. We conclude that water ice in disks originates from the earliest phases of star formation, providing the missing link between cold dark clouds and (exo-)comets.

## Abstract (web summary)

The origin of water is one biggest unknowns in the field of star and planet formation: is it inherited or created in situ? Through the detection of heavy water (D_2_O) in a protoplanetary disk, it is shown that this water must be older than the central star.

## Main

Water may have been delivered to Earth via cometary and/or asteroid impacts, tracing the pristine material left over from the protoplanetary disk where the Solar System originated^1^. However, it is unclear whether the water ice on these bodies primarily formed in, for example, the protoplanetary disk phase or is much older and originated from the parent molecular cloud^2^. This is because the bulk water reservoir is extremely difficult to detect and follow across the various phases of star and planet formation^3–8^. If the water molecules that formed in the cloud survive the cloud collapse into a protostar with a surrounding envelope and subsequently the formation of a disk, then protoplanetary disks are injected with a rich chemical icy inventory as many complex organic molecules (COMs) have formation pathways in the ice (for example, refs. ^9–11^). This potential ice inheritance would set the initial conditions for the chemical inventory of protoplanetary disks and is crucial to interpreting observations of COMs in disks. Intriguingly, if the water ice survives the journey from cloud to disk to comets and possibly (exo-)planets, then the water seen across all these phases and possibly on Earth predates the formation of the star and our Sun, respectively^2^.

A sensitive tracer to distinguish the inheritance and (partial) reset of water ice from pre-stellar cores to protoplanetary disks is the deuteration level of water. In case of reset, at least one of the chemical bonds between the atoms of the water molecule is destroyed. The deuteration is enhanced only above the D/H ratio of 2 × 10^−5^ in the interstellar medium (ISM)^12^ under specific conditions: below 25 K and where the density is sufficiently high for the freeze-out of atoms and molecules (few times 10^4^ cm^−3^; for example, refs. ^13,14^); therefore, water ice formed at these low temperatures will be rich in deuterated water isotopologues. On the contrary, reprocessing of this pristine material at temperatures higher than 500 K can lower the abundance of deuterated water isotopologues by destroying the molecules themselves^15–17^. The high level of singly deuterated water (HDO) seen in protostellar envelopes, disks and comets has been presented as evidence of the inheritance of water (refs. ^14,18,19^, and references therein). However, the abundance of HDO can be as high as ~10^−3^ with respect to H_2_O even if the majority of the water reservoir is reprocessed^17^. Instead, doubly deuterated water (D_2_O) is the most sensitive tool to distinguish inheritance from reset because it cannot reform efficiently after reprocessing. Yet, observations targeting this isotopologue are rare across all star- and planet-formation phases because its abundance is expected to be low even if deuteration is enhanced because of the double deuteration and the low D/H of the ISM.

In particular, observations of doubly deuterated water are missing in the protoplanetary disk stage. The few existing observations probing water ice in disks cannot distinguish whether water is formed in the initial stages of star and planet formation or in situ due to a lack of detected isotopologues (for example, refs. ^20,21^). In addition, most protoplanetary disks are too cold to host a large and observable reservoir of gas-phase water. Tracers other than water isotopologues provide evidence for both inheritance and reprocessing depending on the disk and tracers observed. Inheritance is suggested by the detection of gas-phase methanol (CH_3_OH) in three disks that are too warm for substantial CO freeze-out^22–26^. As CH_3_OH can only form efficiently when CO is frozen-out, the CH_3_OH ice in those disks must have formed before the envelope collapsed to form a disk and survived this process^10,27^. However, the deuteration seen in CH_3_OH and other COMs in the same disk as the one analysed in this work, V883 Ori, is lower than expected based on a much younger protostellar envelope and a much older comet. This has been interpreted as an indication for some reprocessing of the ices in this disk^28^, yet possibly CH_3_OH is slightly more reprocessed than water due to its lower sublimation temperature^29^. We present observations in the V883 Ori disk of doubly deuterated water D_2_O, the most sensitive tracer to distinguish inheritance from reprocessing, to shed light on the origin of ices in disks, in particular the origin of water ice.

## Results

We detect the *p*-D_2_O 1_1,0_–1_0,1_ transition at 316.7998 GHz in the disk surrounding the young, outbursting V883 Ori star with the Atacama Large Millimeter/submillimeter Array (ALMA). This star is located at 400 pc (1300 ly) in the Orion molecular cloud^30^ and has a source velocity of 4.3 km s^−1^ (refs. ^31,32^). Freshly sublimated ices are seen in this disk due to the heating from the outbursting star allowing for a unique look into the bulk water reservoir^18,32,33^. We compare our D_2_O detection with the previously observed HDO and H_2_^18^O emission in this disk^18^.

**Fig. 1:**
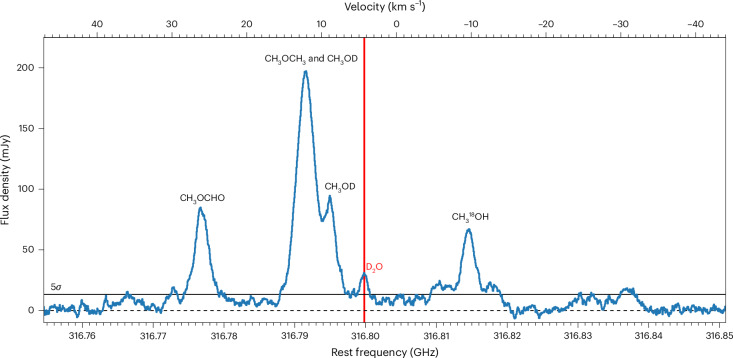
Integrated spectrum of D_2_O in the V883 Ori disk. The spectrum is extracted from an elliptical region with a 0.4″ semi-major axis and a 0.4″ × cos(*i*) semi-minor axis centred on the continuum peak, with *i* the disk inclination. The pixels in the image cube were shifted by the projected Keplerian velocity at that location in the disk before extracting the spectrum to correct for the projected Keplerian rotation of the disk and decrease line blending of D_2_O, indicated with the red vertical line, with the neighbouring CH_3_OD and CH_3_OCH_3_ lines. The solid horizontal line indicates the 5*σ* noise level measured in off-source regions.

**Extended Data Fig. 1:**
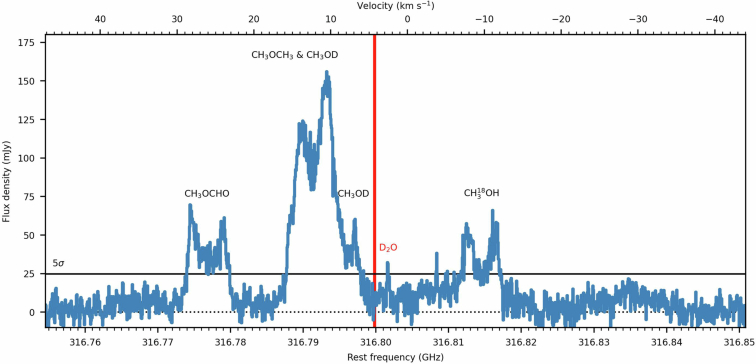
Integrated spectrum of D_2_O in the V883 Ori disk extracted from a 0.4″ × 0.31″ (semi-axes) region. The red vertical line indicates the center of the double peaked D_2_O emission due to the Keplerian rotation of the disk. Other bright emission lines are indicated in black. The solid horizontal line indicates the 5*σ* noise level measured in off-source regions in the image cube.

**Extended Data Fig. 2:**
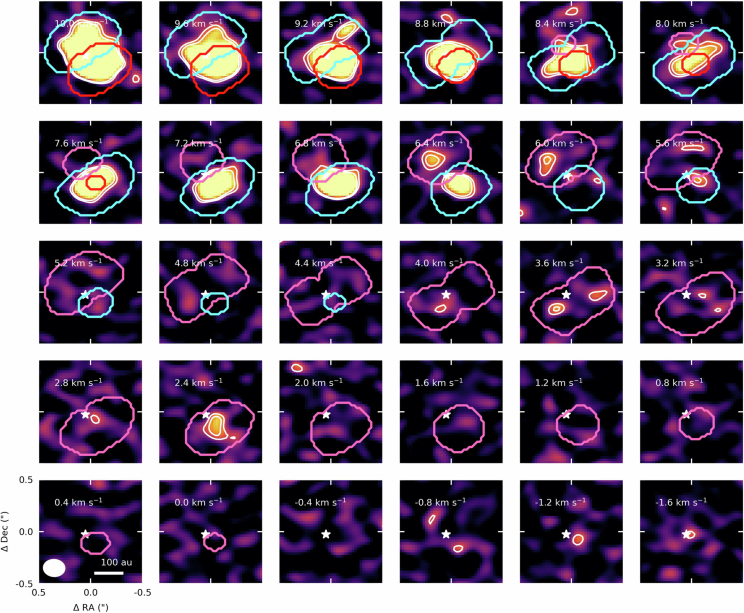
Channel maps of the D_2_O emission. The pink contour indicates the region where D_2_O is expected to emit if the emission is Keplerian. The white contours indicate the 3 and 4*σ* level for the emission of D_2_O and the neighbouring COMs. The cyan and red contours indicate the Keplerian mask where the neighboring lines that are blended with D_2_O are expected to emit.

**Extended Data Fig. 3:**
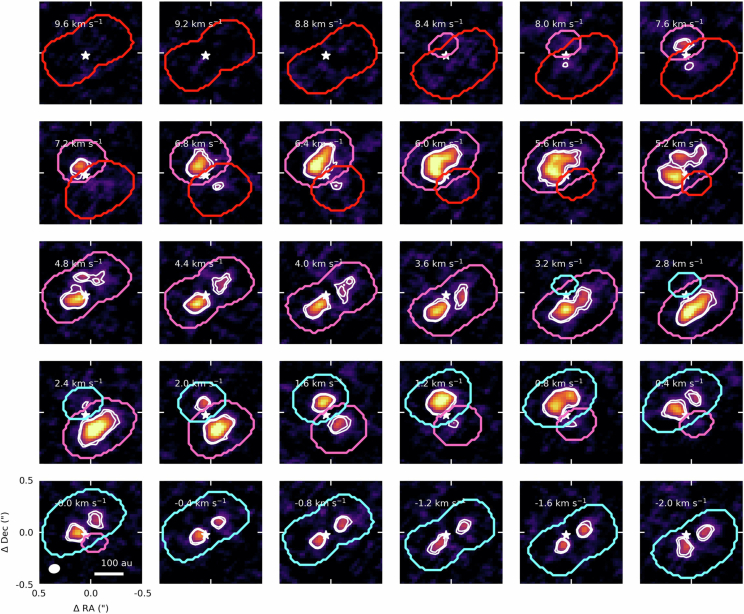
Channel maps of the HDO emission. The pink contour indicates the region where HDO is expected to emit if the emission is Keplerian and the white contours indicate the 3 and 4*σ* level in these channels. The cyan and red contours indicate the Keplerian mask where the neighboring lines that are blended with HDO are expected to emit.

**Extended Data Fig. 4:**
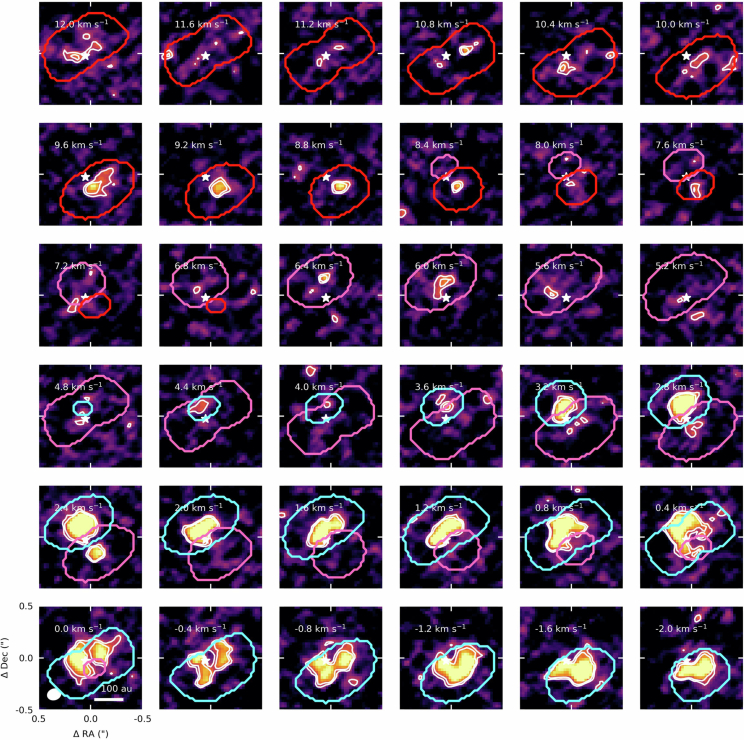
Channel maps of the H_2_^18^O emission. The pink contour indicates the region where H_2_^18^O is expected to emit if the emission is Keplerian and the white contours indicate the 3 and 4*σ* level in these channels. The cyan and red contours indicate the Keplerian mask where the neighboring lines that are blended with H_2_^18^O are expected to emit.

**Extended Data Fig. 5:**
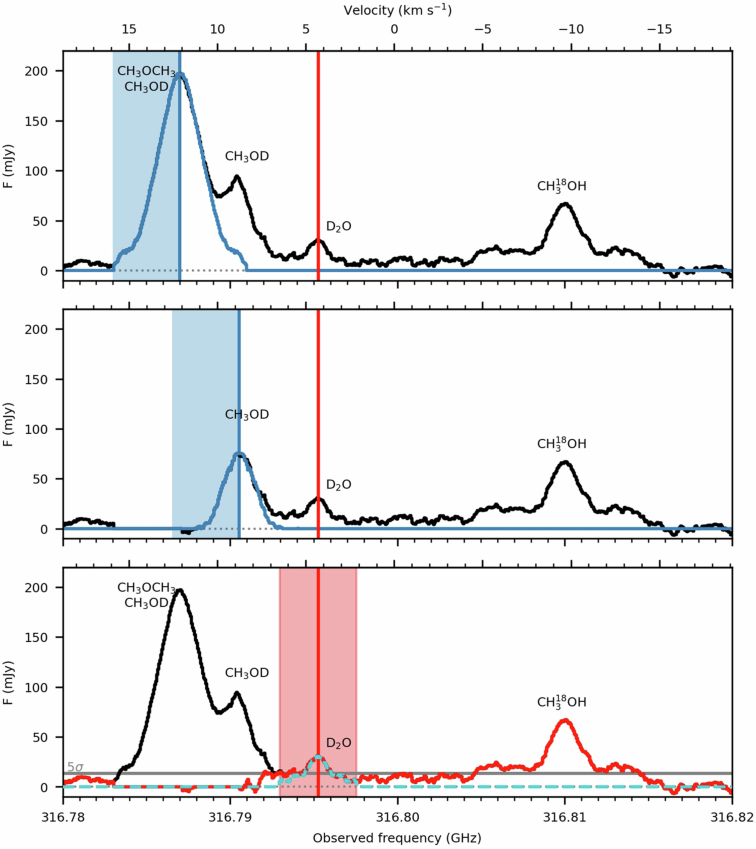
D_2_O flux measurement using the shifted spectra. The lines blended with the D_2_O line are subtracted by extracting the line profile of the clean, low frequency side of the blend (blue shaded region), mirroring this in the frequency of the line and subtracting. The top panel shows the model for the CH_3_OD and CH_3_OCH_3_ in blue. In the middle panel, this model is subtracted from the data (black) and the new model for the CH_3_OD is shown in blue. The D_2_O line is indicated with the red vertical line in the bottom panel. The cyan line profile in this panel is used to measure the D_2_O flux.

**Extended Data Fig. 6:**
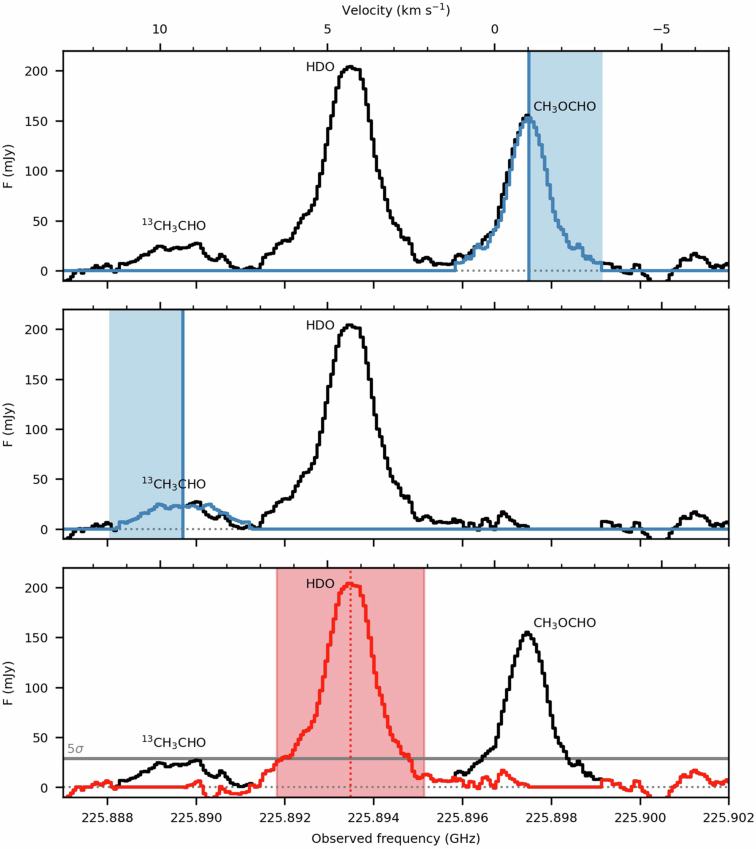
HDO flux measurement using the shifted spectra. The lines blended with the HDO line are subtracted by extracting the line profile of the clean, low frequency side of the blend (blue shaded region), mirroring this in the frequency of the line and subtracting. The top panel shows the model for the CH_3_OCHO in blue. In the middle panel, this model is subtracted from the data (black) and the new model for the ^13^CH_3_CHO is shown in blue. The HDO line is indicated with the red vertical line in the bottom panel.

**Extended Data Fig. 7:**
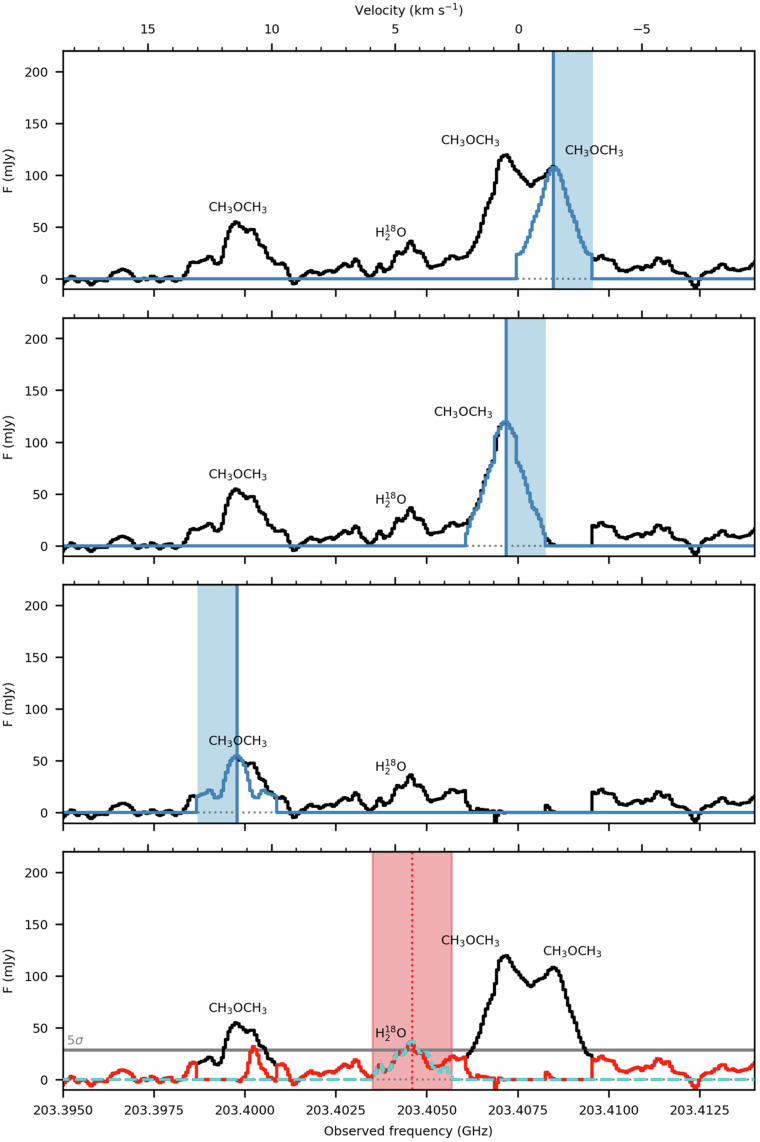
H_2_^18^O flux measurement using the shifted spectra. The lines blended with the H_2_^18^O line are subtracted by extracting the line profile of the clean side of the blend (blue shaded region), mirroring this in the frequency of the line and subtracting. The top three panels demonstrate this process for three CH_3_OCH_3_ lines. The cyan line profile in the bottom panel is used to measure the H_2_^18^O flux.

**Extended Data Fig. 8:**
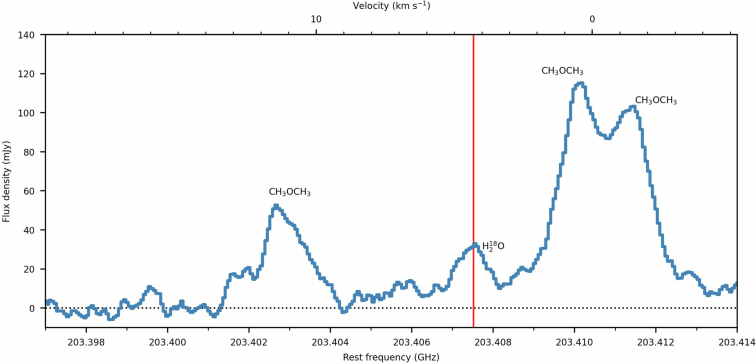
Shifted spectrum of the H_2_^18^O line extracted from an elliptical region with a semi-major axis of 0.35″. This spectrum clearly shows that the H_2_^18^O line is detected in this disk.

The D_2_O line observed in this programme is blended with emission from neighbouring transitions of deuterated methanol (CH_3_OD) at 316.7916 GHz (3_2,1_–3_−1,3_ A) and 316.7951 GHz (7_0,7_–6_0,6_ A) and 2 dimethyl ether (CH_3_OCH_3_) transitions at 316.7915 GHz (22_6,16_–22_5,17_ EE) and 316.7925 GHz (22_6,16_–22_5,17_ AA), respectively, similar to observations of younger class 0 objects^34^. To separate the D_2_O from these COMs, each pixel in the image cube is shifted by the projected Keplerian velocity associated with that pixel to remove the rotation component of the line velocity, before extracting the disk integrated spectrum in an elliptical region with a 0.4″ semi-major axis, presented in Fig. 1. The non-shifted version is presented in Extended Data Fig. 1. The D_2_O line is detected at a peak signal-to-noise ratio of 11 in the shifted spectrum, where the noise is estimated using the root mean square of 520 independent shifted spectra extracted from a 19" square in the cube without primary beam correction, excluding the inner 2.4″ square to avoid possible contamination with extended line emission. In addition, the channel maps show significant emission across multiple channels that can only be attributed to the D_2_O molecule and not to any neighbouring lines of COMs. An overview of the D_2_O, HDO and H_2_^18^O line properties is presented in Table 1. The line flux is measured both from the channel maps presented in Extended Data Figs. 2–4 and from the shifted spectra presented in Extended Data Figs. 5–8. An overview of these line fluxes is presented in Table 2, where the re-imaged HDO and H_2_^18^O emission originally presented in ref. ^18^ are included for consistency. The flux measured using the Keplerian masks is considered to be the fiducial flux (see ‘Flux measurements’ in Methods for details).

**Table 1:** Properties of the transitions analysed in this work

Molecule	Transition	Frequency (GHz)	log *A*_ul_ (s^−1^)	*E*_u_ (K)	*g*_u_	*Q* (300 K)	*Q* (150 K)	*Q* (75 K)
D_2_O	1_1,0_–1_0,1_	316.79981	−3.20	32.6	3	349.6	123.7	44.4
HDO	3_1,2_–2_2,1_	225.89672	−4.88	167.6	7	146.9	52.3	18.9
H_2_^18^O	3_1,3_–2_2,0_	203.40752	−5.31	203.7	7	179.6	64.2	23.4

The D_2_O line constants are taken from the Cologne Database for Molecular Spectroscopy database^62–67^ and those for HDO and H_2_^18^O are taken from the Jet Propulsion Laboratory database^62,68,69^. The line constants for D_2_O and H_2_^18^O use an *ortho*-to-*para* ratio of 2 and 3, respectively.

**Table 2:** Disk integrated line fluxes excluding emission from neighbouring complex organics

Molecule	Transition	Line flux (mJy km s^−1^)	Line flux (mJy km s^−1^)
		Keplerian mask	Shifted spectrum
D_2_O	1_1,0_–1_0,1_	53 ± 4	61 ± 4
HDO	3_1,2_–2_2,1_	358 ± 14	408 ± 12
H_2_^18^O	3_1,3_–2_2,0_	43 ± 9	58 ± 10

The HDO and H_2_^18^O line fluxes are a reanalysis of those originally presented in ref. ^18^. The uncertainty on the flux does not include the absolute flux calibration uncertainty of ALMA. The line fluxes are measured between 1.2 km s^−1^ and 6.4 km s^−1^ for the Keplerian masking method.

**Fig. 2:**
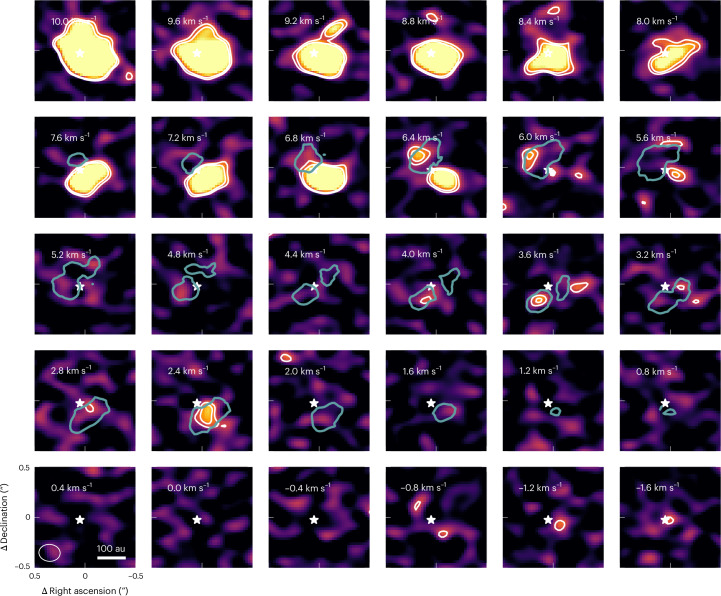
Channel maps of the D_2_O emission compared with that of HDO. The velocity of each channel is indicated by the white text in the top left corner of each panel. The grey line indicates the emission above 3*σ* attributed to the HDO line at 225 GHz. The white contours indicate the 3*σ* and 4*σ* level for the emission of D_2_O and the neighbouring COMs.

As the spatial distribution of all 3 water isotopologues (D_2_O, HDO and H_2_^18^O) is similar (see Fig. 2 for the channel maps of D_2_O and HDO and ref. ^18^ for a comparison of HDO and H_2_^18^O), we use the excitation temperature of 199 ± 42 K of the HDO molecule^18^, the only water isotopologue with 2 observed transitions, for all the water isotopologues. The effect of the uncertainty on this excitation temperature is discussed in Supplementary Information, section 3.1 as one of the HDO lines used for this measurement is heavily blended with an emission line of a COM, possibly affecting the derived excitation temperature. The line fluxes are measured using Keplerian masks that separate the water isotopologue emission from that of the neighbouring COMs in right ascension, declination and velocity. These line fluxes of the D_2_O, HDO and H_2_^18^O transitions are then converted to column densities *N* of (4.2 ± 1.2) × 10^13^ cm^−2^, (49.5 ± 6.9) × 10^14^ cm^−2^ and (23.7 ± 5.7) × 10^14^ cm^−2^, assuming an emitting area equal to the elliptical region used to extract the spectrum and the flux integrated over the velocity channels where all 3 water isotopologues can be separated from other emission lines (see also ‘Flux measurements’ and ‘Column density estimates’ in Methods, Table 3 and Supplementary Information, section 3). As no strong isotope selective effects are expected between H_2_O and H_2_^18^O, the H_2_^18^O column density is scaled to a total H_2_O column density of (13.3 ± 3.2) × 10^17^ cm^−2^ using the typical ^16^O/^18^O ratio of 560 ± 25 in the ISM^35^, consistent with the lower limit derived in the HL Tau disk of *N*(H_2_^16^O)/*N*(H_2_^18^O) > 40 (ref. ^36^). These column densities translate to D_2_O/H_2_O = (3.2 ± 1.0) × 10^−5^ and (D_2_O/HDO)/(HDO/H_2_O) = 2.3 ± 1.0.

**Table 3:** Column densities and column density ratios of water isotopologues

Method	*N*(D_2_O)	*N*(HDO)	*N*(H_2_^18^O)	D_2_O/H_2_O	D_2_O/HDO	$\frac{{{\mathbf{D}}}_{\mathbf{2}}{\mathbf{O}}/{\mathbf{HDO}}}{{{\mathbf{HDO}}/{\mathbf{H}}}_{\mathbf{2}}{{\mathbf{O}}}}$
	(10^13^ cm^−2^)	(10^14^ cm^−2^)	(10^14^ cm^−2^)	(×10^−5^)	(×10^−3^)	
Keplerian mask	4.2 ± 1.2	49.5 ± 6.9	23.7 ± 5.7	3.2 ± 1.2	8.5 ± 2.8	2.3 ± 1.0
Shifted spectrum	4.9 ± 1.4	56.4 ± 7.7	32.3 ± 6.3	2.7 ± 1.0	8.6 ± 2.8	2.8 ± 1.1

All uncertainties include the absolute flux calibration uncertainty of ALMA, the uncertainty on the assumed excitation temperature and the uncertainty on the ^16^O/^18^O ratio.

## Discussion and conclusion

### Water isotopologue ratios along the water trail

**Fig. 3:**
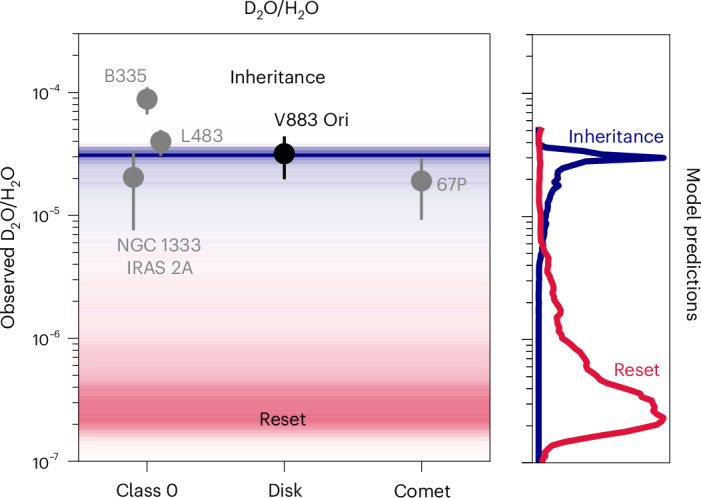
The D_2_O/H_2_O ratio across different stages of star and planet formation. The measurements in the V883 Ori disk are presented in black and those in the class 0 objects NGC 1333 IRAS 2A, B335, L483 and the 67P comet in grey^34,37–40^. The latter are computed as D_2_O/HDO × HDO/H_2_O. The error bars represent the 1*σ* uncertainty (s.d.) on the measured column density ratio in each source. The coloured background and the histograms on the side, each normalized to the peak number of fluid parcels, indicate the expected water isotopologue ratios for inheritance where ≲10% of the H_2_O ice is destroyed (blue) or reset where ≳70% of the H_2_O is expected to be destroyed through photodissociation and photodesorption in a model of a collapsing core^17^ (red). The red histogram is smoothed using a Savitzky–Golay filter with a window of 10 and an order of 3.

Both the D_2_O/H_2_O and the (D_2_O/HDO)/(HDO/H_2_O) ratios in the V883 Ori disk are similar to the observed values of D_2_O/H_2_O = (2–9) × 10^−5^ and (D_2_O/HDO)/(HDO/H_2_O) = 2.2–7.1 in younger class 0 objects^34,37,38^, which are expected to trace pristine ices. In addition, the D_2_O/H_2_O ratio in the V883 Ori disk is similar to that of (1.9 ± 1.0) × 10^−5^ in the comet 67P (refs. ^39,40^ and Fig. 3). This strongly indicates that the ice in the V883 Ori disk is inherited from the cold molecular cloud that collapsed to form a class 0 protostar with protostellar envelope and subsequently an embedded disk, and potentially connects to the cometary phase.

The D_2_O abundance in the V883 Ori disk is not expected to be greatly altered by the outburst as modelling of HDO and H_2_O in a one-dimensional evolving disk around an outbursting star has shown that the outburst affects only the water deuteration in the inner 1–3 au (refs. ^15^). The emission of the water isotopologues in the V883 Ori disk is only seen much farther out at radii larger than 40 au, as optically thick dust hides the line emission inside that radius^18,31^.

The coloured background in Fig. 3 indicates the range of D_2_O/H_2_O ratios consistent with inheritance (blue) and reset (red) based on chemical modelling of gas and ice in a collapsing core by ref. ^17^. These simulations follow gas parcels as the rotating core collapses from the inside out and forms a disk and envelope. These stream lines are post processed with a dedicated chemical network modelling the deuterium chemistry in the gas, a chemically active surface layer of ice interacting with the gas and a chemically inert bulk ice phase. As these stream lines trace a range in physical conditions, the water isotopologue ratios also show a range of possible values for either inheritance (all stream lines where ≲10% of the water ice is destroyed) or reset (all stream lines where ≳70% of the water ice is destroyed). The V883 Ori datapoint coincides with the peak of the model prediction histogram for inheritance well within 1*σ*, further supporting that the material in this disk is inherited.

**Extended Data Fig. 9:**
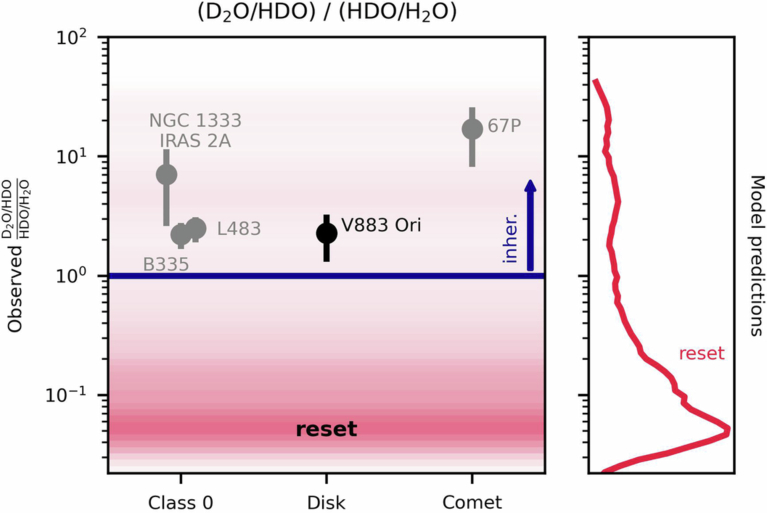
The (D_2_O/HDO)/(HDO/H_2_O) ratio across different stages of star and planet formation. The measurements in the V883 Ori disk are presented in black and those in the class 0 objects NGC 1333 IRAS 2A, B335, L483, and the 67P comet in grey^34,37–40^. The errorbars represent the 1*σ* uncertainty (s.d.) on the measured column density ratio in each source. The colored background and the histograms each normalized to the peak number of fluid parcels on the side indicate the expected water isotopologue ratios for reset where ≳ 70% of the H_2_O is expected to be destroyed through photodissociation and photodesorption in a model of a collapsing core (red;^17^). The red histogram is smoothed using a Savitzky-Golay filter with a window of 10 and an order of 3. The horizontal blue line and arrow indicate an approximate boundary between inheritance ((D_2_O/HDO)/(HDO/H_2_O) ≳ 1) and reset ((D_2_O/HDO)/(HDO/H_2_O) ≲ 1)^17^.

The observed (D_2_O/HDO)/(HDO/H_2_O) ratio is consistent between class 0 objects and the V883 Ori disk and are all a factor of ~40–140 higher than the most likely value of ~5 × 10^−2^ in the case the material is reset (Extended Data Fig. 9). The (D_2_O/HDO)/(HDO/H_2_O) ratio predicted in ref. ^17^ for inheritance is 10, which is a factor up to ~5 higher than that observed. Nonetheless, in contrast to the values for reset, the initial (D_2_O/HDO)/(HDO/H_2_O) can vary between ~0.8 and ~19 due to variations in the initial conditions of the models^16^ (see Supplementary Information for details). The similarity in the observed ratio between the V883 Ori disk and the class 0 objects strongly suggests that material is inherited.

As all 3 analysed transitions lie at different frequencies of 316.7998 GHz (D_2_O), 225.8967 GHz (HDO) and 203.4075 GHz (H_2_^18^O), frequency-dependent radiative transfer effects could affect the observed D_2_O/H_2_O and the (D_2_O/HDO)/(HDO/H_2_O) ratios. In particular, the dust inside 40 au is optically thick^31,41^ absorbing the emission of COMs and other molecules whose emission partly originates at altitudes comparable to the optically thick layer of the continuum emission^18,28,33,42,43^. As the continuum optical depth increases with frequency, more D_2_O emission could be hidden than HDO and H_2_^18^O due to the higher frequency of the D_2_O line. Therefore, if optically thick dust is affecting the lines differentially, the measured ratios will be driven further into the regime consistent with inheritance. Other systematics that could affect the measured D_2_O/H_2_O and (D_2_O/HDO)/(HDO/H_2_O) ratios such as a lower excitation temperature are discussed in Supplementary Information, section 3.

### Deuteration of COMs

The high abundance of D_2_O with respect to H_2_O in the V883 Ori disk demonstrates that water in this disk is probably inherited. This is further supported by the high gas-phase HDO/H_2_O ratio seen across protostellar envelopes, disks and comets (ref. ^18^, and references therein) and the high HDO/H_2_O ratio seen in the ice in a single low-mass protostar and two massive protostars^19,44^. However, the abundance of CH_2_DOH, CH_3_CDO, CH_3_OCDO and CH_2_DOCHO compared with their main isotopologues is measured to be lower in the V883 Ori disk than in the younger hot corino IRAS 16296 B and the comet 67P. This lower level of deuteration in the V883 Ori disk has been interpreted as an indication for some reprocessing of COMs^28,45^.

COMs are traditionally thought to form in CO-rich ice but there are indications in the V883 Ori disk that COMs possibly partially formed in a water-rich ice matrix (H_2_O)^45^. The contribution from COMs formed in the CO-rich ice matrix is expected to have a higher D/H ratio as deuteration is efficient at the temperature where CO freezes-out. The initial deuteration levels of these COMs probably follows the D_2_O/HDO ratio as D_2_O and HDO are formed at the same time. The levels of deuteration seen in a CH_2_DOH/CH_3_OH ratio of 1.97 × 10^−2^ (refs. ^33,45^) and the low upper limits on the deuteration in CH_2_DOH, CH_3_CDO, CH_3_OCDO and CH_2_DOCHO of ≲(5.7–20) × 10^−3^ (ref. ^28^) are consistent with the D_2_O/HDO ratio of (8.5 ± 2.8) × 10^−3^ in this disk. Yet, chemical reactions where H and D are exchanged or abstracted can alter the D/H ratio in COMs, complicating the analysis (for example, ref. ^46^). Even though COMs are likely to at least partially form in the CO-rich ice matrix, the contribution of COMs formed within the water-ice matrix will have a low deuteration level due to the higher gas temperatures at the time of formation making deuteration inefficient.

### Summary

The inheritance of water from the ice on dust grains in the cloud before stars are born to the disk and subsequently to comets connects the major steps in the formation of planets as comets probably form from the same reservoir of material as the planets. Our result demonstrates that pristine water that formed at the earliest phases of star and planet formation is available in protoplanetary disks, a phase where potential hints of embedded planets are frequently seen in the form of, for example, dust substructures (for example, ref. ^47^). Even though the journey of water from the disk to Earth is still debated and the deuteration in Earth’s oceans is lower than the HDO/H_2_O ratio in the V883 Ori disk^1,18^, the deuteration of Earth’s oceans is enhanced compared with the ISM by an order of magnitude^12,48,49^. In addition, the D_2_O/H_2_O ratio is similar across class 0 objects, the V883 Ori disk and a comet. Together with the enhanced deuteration seen in HDO across all these four phases, this suggests that the trail of inherited water does not stop at the cometary phase but potentially continues to the water present on exoplanets formed in water-ice-rich protoplanetary disks.

## Methods

### Self-calibration

We observed the V883 Ori disk in ALMA band 7 for 2.4 hours on-source distributed over 3 execution blocks (EBs; Supplementary Table 1; 2023.1.00588.S; principal investigator M.L.). These data targeted the D_2_O 1_1,0_–1_0,1_ transition at 316.7998 GHz at a spectral resolution of 61 kHz (58 m s^−1^) and a total bandwidth of 117.2 MHz. A continuum spectral window covering 1.875 GHz of bandwidth at a spectral resolution of 1.1 km s^−1^ was centred at 315 GHz. In addition, 6 spectral windows centred at the ^13^CN transition with quantum numbers *N* = 3–2, *J* = 5/2–3/2, *F*_1_ = 2–1, *F* = 3–2 (325.943 GHz), N_2_O 13_0,0_–12_0,0_ (326.556 GHz), N_2_O 13_−1,2_–12_1,2_ (326.685 GHz), CH_3_OCH_3_ 18_1,18_–17_0,17_ EE (326.931 GHz), NO_2_ 22_1,21_–220, 22, *J* = 45/2–45/2, *F* = 43/2–43/2 (328.097 GHz), and NO_2_ 22_1,21_–22_0,22_, *J* = 45/2–45/2, *F* = 47/2–47/2 (328.131 GHz) were included with a spectral resolution of 122 kHz (~130 m s^−1^) and a bandwidth of 58.6 MHz. Finally, the CH_3_OCH 10_3,8_–9_2,7_ EE transition at 328.857 GHz was targeted at the same spectral resolution but a bandwidth of 117.2 MHz.

The data were self-calibrated using CASA version 6.5.4^50^ following the analysis used in the exoALMA large program outlined in ref. ^51^, with several routines by refs. ^52,53^. A pseudo continuum measurement set was created for each EB after carefully flagging all spectral regions exhibiting line emission, and then averaging all the data in individual channels for each spectral window. As this is a very line-rich disk, the total continuum bandwidth used for the self-calibration was reduced to 180 MHz. Before aligning and then combining the three EBs, one round of phase self-calibration over a solution interval equal to the length of a single EB (1 hour and 19 minutes) was performed combining all scans and both polarizations. A CLEAN model was used for the self-calibration, as constructed over an elliptical mask of 0.6″ in semi-major axis, and a position angle and semi-minor axis computed from the position angle and inclination of the V883 disk of 32° and 38.3°, respectively^31^. We employed Briggs weighting with robust = 0.5. The model was created by cleaning the emission in all line-free channels down to a conservative threshold of 6*σ*. The peak signal-to-noise ratio in the images of the individual EBs improved between 76% and 160%. The continuum observed in the third EB had the highest signal-to-noise ratio of all EBs and therefore was used as a reference for the alignment where the phase angle and the amplitude differences are minimized between the first EBs and the third EB^51^.

After combining all execution blocks, 7 rounds of phase-only self-calibration were performed with solution intervals with the length of a single EB, 360, 120, 60, 20, 10 and 6 seconds. The imaging followed the same mask and weighting as for the individual EBs before concatenation. In the first round of phase self-calibration, separate solutions were found for both polarizations, whereas in all subsequent rounds, a single solution was obtained for both polarizations. In addition, all scans were combined during the first three rounds as the solution interval exceeded the scan length.

Comparing the amplitude of the continuum visibilities between the three execution blocks showed that the data taken on 24 December 2023 had an 8.6% higher amplitude across all baselines up to 600 k*λ*, with *λ* the wavelength of the observations, where the signal-to-noise ratio drops compared with the execution block with the highest signal-to-noise ratio. Therefore, we rescaled the flux before self-calibration in this execution block and concatenated this to the data of the other two EBs before self-calibration. Subsequently, these concatenated data were self-calibrated with seven rounds of phase-only self-calibration and a single round of phase and amplitude self-calibration with separate solutions for both polarizations and a solution interval of the length of a single EB. The model for the amplitude self-calibration was created by cleaning the line-free channels down to a 1*σ* threshold to capture as much emission as possible in the model. Solutions deviating by more than ~20% in amplitude were flagged. The phase self-calibration improved the peak signal-to-noise ratio of the data by a factor of 186%, from a peak signal-to-noise ratio of 495 to one of 1,457. The amplitude and phase self-calibration improved the peak signal-to-noise ratio by 3% (see Supplementary Fig. 1 for the final continuum image). The peak continuum intensity increases by 15% after phase-only self-calibration due to the improved phase coherence after phase-only self-calibration. The peak continuum intensity changes by less than 0.5% after phase and amplitude self-calibration. The solutions were then applied to the full dataset, following the same logical order as for the pseudo continuum measurement set.

### Continuum subtraction

As many lines of COMs are detected in this dataset, too few line-free channels were available to subtract the continuum emission in the *uv*-plane using the standard CASA task uvcontsub. Instead, the continuum in the D_2_O spectral window was subtracted in each EB separately. First, a mtmfs image up to and including first order (nterms = 2) was created using the line-free channels in the D_2_O spectral window. The resulting model for the continuum emission was added to the measurement set using ft. This model was then used to subtract the continuum using the CASA task uvsub. The resulting MS tables for all three EBs were then combined to make the final continuum-subtracted dataset for D_2_O.

### Imaging

The D_2_O line itself was imaged using the CASA task tclean with Briggs weighting and a robust parameter of 0, which provides the best balance between spatial resolution and noise, resulting in a 0.24″ × 0.19″ (89°) beam. The spectrum around the D_2_O emission is presented in Extended Data Fig. 1 and the D_2_O channel maps are presented in Fig. 2. The relatively small beam allows for better deblending of the lines while still detecting the line. The image is cleaned down to 1*σ* to capture all the flux in the model and make the final measurement independent of any non-Gaussian features in the beam at the cost of potential imaging artefacts^53,54^. The channel spacing for the final image is 400 m s^−1^ to match that of the HDO and H_2_^18^O data. The resulting cubes are used to produce channel maps and measure the flux using a Keplerian mask. Only when the data cubes are shifted with the projected Keplerian velocity associated with each pixel, the native spectral resolution of the data of 58 m s^−1^, 162 m s^−1^ and 180 m s^−1^ for D_2_O, HDO and H_2_^18^O, respectively, is used to minimize artefacts due to the finite spectral resolution of the data.

To accurately measure the (D_2_O/HDO)/(HDO/H_2_O) and D_2_O/H_2_O ratio in this disk, we re-imaged the HDO and H_2_^18^O data presented in ref. ^18^ (2021.1.00186.S; principal investigator J.J.T.). In this work, these data are cleaned down to 1*σ*, similar to the D_2_O line, and allow for a uniform analysis of the line fluxes and mitigate any effects of the non-Gaussian beam shape on the measured line flux (for example, ref. ^53^). The resulting beam sizes are 0.13″ × 0.11″ (−72°) and 0.10″ × 0.08″ (−78°) for H_2_^18^O and HDO when cleaned with a robust parameter of 1.0 and 2.0, respectively, to provide the best balance between the signal-to-noise ratio and a small beam to separate the lines from neighbouring COMs. The resulting channel maps for HDO and H_2_^18^O are presented in Extended Data Figs. 3 and 4, respectively.

### Flux measurements

The transitions of all three water isotopologues discussed in this paper are blended with lines from neighbouring COMs. To separate these lines and accurately measure the line flux, two methods are employed: Keplerian masking in the channel maps and spectral shifting of the spectrum. In the former case, the lines are separated in the image plane by computing the region in right ascension and declination where a molecule is expected to emit based on the Keplerian rotation of the disk and its orientation on the sky. As this region shifts for each velocity channel, lines of neighbouring COMs can be separated from the water isotopologue emission. The Keplerian masks are constructed using the package by ref. ^55^ following ref. ^18^ using a distance of 400 pc (ref. ^30^), a stellar mass of 1.3 *M*_⊙_, a disk inclination of 38.3° and a position angle of 32° (ref. ^31^) and an emitting region extending from 40 au to 120 au tracing a height to radius ratio of *z*/*r* = 0.4. The line width is assumed to follow $350\,{\rm{m}}\,{{\rm{s}}}^{-1}\times \sqrt{40\,{\rm{au}}/r}$ and the mask is shifted by 4.3 km s^−1^ to match the systemic velocity of V883 Ori^31,32^. These parameters are identical between all three analysed water isotopologues. Finally, the mask is convolved with a Gaussian 0.5 (D_2_O), 1.25 (HDO) and 0.75 (H_2_^18^O) times larger than the beam of the respective observations. This parameter is chosen after visual inspection of the data. The factor varies slightly from line to line to match the spatial extent of the emission observed in the channel maps and to match the spatial resolution of the resulting masks to have an ~0.1−0.12″ major axis and a 0.08−0.1″ minor axis across all lines. The effect of neighbouring COMs is investigated using an identical mask but then shifted to the frequency of those lines.

In general the emission of the D_2_O, HDO 225 GHz and the H_2_^18^O transition can be well separated in most channels using this method. To measure the disk integrated flux, only the channels between 1.2 km s^−1^ and 6.4 km s^−1^ are used as only in these channels the water emission can be separated from the COMs for all three isotopologues. The uncertainties on the disk integrated line fluxes are estimated as the root mean square of the flux measured in masks shifted to 8 different positions 1.4″ from the original location and to 5 different velocities resulting in 40 unique, non-overlapping locations. The HDO 241 GHz line, however, is too severely blended and is therefore not considered in this analysis.

The second method to deblend the lines is through spectral shifting. Similar to the Keplerian masking, this method utilizes the Keplerian rotation of the disk. Each pixel is shifted to correct for the projected Keplerian velocity expected at this location in the disk. The lines in the spectrum extracted from such a shifted cube are much narrower and single peaked than the double-peaked line profiles in the non-shifted cube. As the lines are only seen over a small radial range, the emission is assumed to be originating from the disk midplane.

The resulting line profiles are not necessarily Gaussian. Therefore, the line profile of each COM is reconstructed from the data directly (Extended Data Figs. 5–7). The non-blended side of COM emission line is mirrored in the line centre, creating a symmetric line profile that is then subtracted from the shifted spectrum. This procedure is repeated for all bright lines of COMs around the water lines. The D_2_O and H_2_^18^O lines were still blended by some remaining COM emission. Therefore, a spectral template for the D_2_O and H_2_^18^O themselves were created using the clean side of the line after the COM emission was subtracted (see the cyan line in the bottom panel of Extended Data Figs. 5 and 7). For H_2_^18^O, the contribution of the contaminating shoulder is negligible at 1*σ*, whereas for D_2_O, the CH_3_OD contributed 8 mJy km s^−1^ to the total D_2_O flux. The region used to create the line profile of the COMs is indicated with the blue shaded region and that used to measure the water isotopologue line flux is indicated in red.

Extended Data Fig. 8 presents a spectrum of the H_2_^18^O line but then extracted from a somewhat smaller region of 0.35″ × 0.27″ (semi-axes). The noise is estimated from 720 independent spectra covering a 19" region in the image plane while excluding the central 2.1″ square in the non-primary beam-corrected cube. As less noise is added to this spectrum than in Extended Data Fig. 7, it clearly shows an emission peak at the expected frequency for the H_2_^18^O line, showing that this line is detected in this dataset. This spectrum is not used for any of calculations to avoid a bias due to the smaller region used to extract this spectrum compared with that of the D_2_O and HDO lines.

An overview of the measured fluxes is presented in Table 2, where for the D_2_O and H_2_^18^O emission, we report the flux measured by the spectral template centred on those lines to account for the remaining shoulder of COM emission in the spectrum. The reported uncertainties on the line fluxes do include the statistical uncertainty but not the 5% absolute flux calibration uncertainty for ALMA Band 5 and Band 6 (HDO and H_2_^18^O) and 10% for ALMA Band 7 (D_2_O). The absolute flux calibration uncertainty is included for all line ratios together with the uncertainty on the ^16^O/^18^O ratio of 560 ± 25 in the ISM^35^. The uncertainty on the ^16^O/^18^O ratio in the ISM is negligible for the resulting H_2_O column density and water isotopologue ratios.

The two methods to measure the line fluxes are consistent within ≤3*σ* for all three water isotopologues. This difference stems from the limited velocity range used for the Keplerian masking method. In this case, only the channels between 1.2 km s^−1^ and 6.4 km s^−1^ are used as all three water isotopologues can be separated from the neighbouring COMs in these channels, whereas emission in some isotopologues is seen up to 0.8 km s^−1^ and 8.4 km s^−1^. As some flux from the inner disk region may be lost due to the spectral shifting of the data as a result of beam smearing and the main goal of this work is to obtain ratios of water isotopologues, the fluxes measured using the Keplerian masks are used for the final analysis presented in the main text.

### Column density estimates

To compute the ratios of water isotopologues, their line fluxes need to be converted to column densities using the properties of the emission lines listed in Table 1. In this work, optically thin emission and local thermodynamical equilibrium are assumed for this conversion. These assumptions are further discussed in Supplementary Information, sections 3.2 and 3.4, respectively. The column density of the upper energy level can be determined without any knowledge on the rotational temperature^56,57^:

1 $${N}_{{\mathrm{u}}}=\frac{4\uppi {F}_{\nu }\Delta V}{{A}_{{\mathrm{ul}}}\varOmega hc},$$

with ∫*F*_*ν*_d*V* ≈ *F*_*ν*_Δ*V* the flux *F*_*ν*_ integrated over velocity *V*, *A*_ul_ the Einstein-A coefficient of the transition, *Ω* the emitting region in steradian, *h* the Planck constant and *c* the speed of light. The water snowline, the midplane region where water sublimates, is possibly at 40 au but most likely around 75–120 au based on gas-phase measurements^18,31,32,58,59^. In addition, gas-phase water traced by HDO and H_2_^18^O is seen out to 160 au (0.4″)^18^ and as the spatial distribution of the D_2_O, HDO and H_2_^18^O water isotopologues is similar (Fig. 2 and Extended Data Figs. 2–4), an elliptical emitting region with that radius is used. The minor axis of this elliptical region is set to 0.4″ × cos(*i*) = 0.31″, with *i* the disk inclination to trace a circular region with a radius of 160 au in the disk frame.

The column density of the upper energy level is then converted to the total column density using the partition function *Q* at the rotational temperature *T*_rot_ and the degeneracy *g*_u_ and the upper energy level *E*_u_ of the line:

2 $${N}_{{\rm{tot}}}=\frac{{N}_{{\mathrm{u}}}Q({T}_{{\rm{rot}}})}{{g}_{{\mathrm{u}}}}\exp \left(\frac{{E}_{{\mathrm{u}}}}{{k}_{{\rm{B}}}{T}_{{\rm{rot}}}}\right),$$

with *k*_B_ the Boltzmann constant. If multiple lines of the same molecule are observed, this equation can be solved for each line to find the excitation temperature of the molecule. In this work, the excitation temperature derived by ref. ^18^ from two HDO transitions with different upper energy levels is used for all analysed lines as these water isotopologues emit from the same disk region.

For the D_2_O and H_2_^18^O molecules that contain two deuterium or two hydrogen atoms, the molecules can be either in an *ortho* or in a *para* state. The transitions used in this work are both *para* states. The column density of *p*-D_2_O and *p*-H_2_^18^O are converted to the total column density using a thermalized *ortho*-to-*para* ratio of 2 and 3, respectively^60,61^. A summary of the derived column densities are presented in Table 3.

## Supplementary information

Supplementary Information Supplementary Figs. 1–3, Tables 1–3, a brief introduction of Supplementary Fig. 1 and Supplementary Table 1, and supplementary water isotopologue ratios and modelling uncertainties.

## Data Availability

The ALMA data from ALMA programmes #2021.1.00186.S and #2023.1.00588.S are publicly available on the ALMA archive.

## Code availability

The code used for this work is based on publicly available Python packages. In addition, the code itself is available upon reasonable request to the corresponding author.
