# Supplementary material for: Pristine ices in a planet-forming disk revealed by heavy water
Source: Nat Astron. 2025 Oct 15;9(10):1486–94. doi: 10.1038/s41550-025-02663-y (PMC12534187; doi:10.1038/s41550-025-02663-y)
Supplement: Supplementary file 1 — Supplementary Figs. 1–3, Tables 1–3, a brief introduction of Supplementary Fig. 1 and Supplementary Table 1, and supplementary water isotopologue ratios and modelling uncertainties. [file 41550_2025_2663_MOESM1_ESM.pdf]

nature astronomy

Supplementary information https://doi.org/10.1038/s41550-025-02663-y

Pristine ices in a planet-forming disk revealed by heavy water

In the format provided by the authors and unedited

Pristine ices in a planet-forming disk revealed by heavy water

Margot Leemker[1*], John J. Tobin[2], Stefano Facchini[1], Pietro Curone[3], Alice S. Booth[4,5], Kenji Furuya[6,7], Merel L. R. van 't Hoff[8]

[1*]Dipartimento di Fisica, Università degli Studi di Milano, Via Celoria 16, Milano, 20133, Italy.
[2]National Radio Astronomy Observatory, Charlottesville, VA, USA.
[3]Departamento de Astronomía, Universidad de Chile, Camino El Observatorio 1515, Las Condes, Santiago, Chile.
[4]Center for Astrophysics, Harvard & Smithsonian, 60 Garden St., Cambridge, 02138, MA, USA.
[6]Department of Astronomy, Graduate School of Science, University of Tokyo, Tokyo, 113-0033, Japan.
[7]RIKEN Pioneering Research Institute, 2-1 Hirosawa, Wako-shi, Saitama, 351-0198, Japan.
[8]Department of Physics and Astronomy, Purdue University, 525 Northwestern Avenue, West Lafayette, 47907, IN, USA.

*Corresponding author(s). E-mail(s): margot.leemker@unimi.it;

Supplementary information

1 Observational details

A full description of the self-calibration of the ALMA data can be found in the Methods section. The dates, number of antennas, configuration, and the bandpass, flux, and phase calibrators are summarized in Supplementary Table 1. In addition, the self-calibrated continuum emission of the V883 Ori disk is presented in Supplementary Figure 1.

Supplementary Data Table 1 ALMA observations covering the D_2O line.

Date	number of antennas	min. baseline (m)	max. baseline (km)	bandpass & flux calibrator	phase calibrator
24 Dec. 2023	43	15.1	1.2	J0423-0120	J0607-0834
28 Jul. 2024	45	15.1	1.4	J0538-4405	J0607-0834
30 Jul. 2024	47	15.1	1.4	J0538-4405	J0607-0834

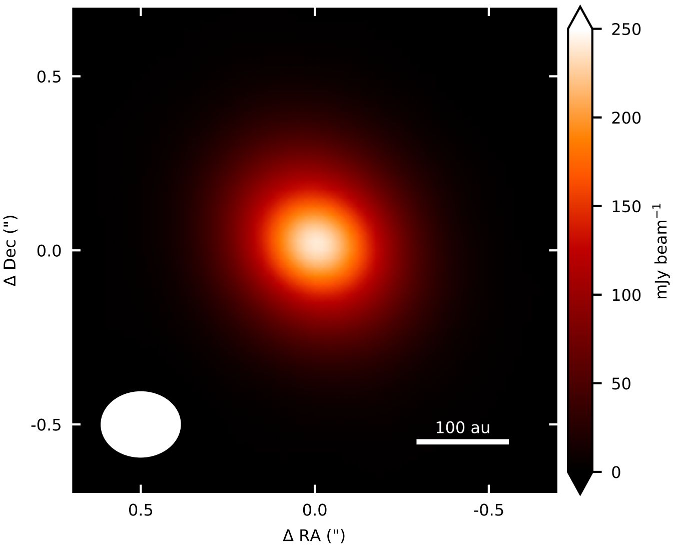

Supplementary Data Figure 1 Continuum image of the V883 Ori disk after self-calibration. The white ellipse in the bottom left corner indicates the beam of the observations.

2 Water isotopologue ratios

The (D_2O/HDO) / (HDO/H_2O) has been suggested as an indicator to distinguish inheritance from reset in addition to the D_2O/H_2O ratio [1]. The HDO/H_2O ratio is more accessible through observations than the (D_2O/HDO) / (HDO/H_2O) ratio as the former only requires observations of two water isotopologues. However, modeling of the ices in a two-dimensional collapsing core shows that the HDO/H_2O ratio in the case of reset can be similar to or even higher than the inherited value due to reformation of HDO ice from deuterium rich fragments on the ice and non-equilibrium gas-phase chemistry whose deuterated products freeze-out [1].

To determine if water is inherited or reset, we follow a twofold argument. First of all, the water isotopologue ratios in the V883 Ori disk are compared to those in younger and older objects to look for similarity. Second, these ratios are compared to the model predictions by [1]. Here, the most important uncertainties in the model predictions are discussed.

2.1 Modelling uncertainties

The rotation of the envelope and number of chemically active monolayers in the chemical network typically only affect the water abundance by a factor of several [1]. As the expected values for D_2O/H_2O and (D_2O/HDO) / (HDO/H_2O) between inheritance and reset generally differ by two orders of magnitude this is not expected to significantly affect the expected ratios.

The deuteration of water is only efficient at sufficiently low temperatures of $\lesssim$ 25 K and densities above a few 10^4 cm^{-3} [2, 3]. Therefore, the deuteration will be more (less) extreme than modelled if the temperature in the V883 Ori system were lower (higher) and the density was higher than in the models by [1].

The abundance of HDO and D_2O in the case of inheritance can be further enhanced if the D/H in the gas increases at the time the water ice forms in the protostellar envelope or protoplanetary disk phase. This higher D/H in the gas only gets transferred to the water ice if the timescale for the gas-phase ion-neutral chemistry is faster than the timescale of the freeze-out of atomic oxygen. These timescales roughly match for a gas density of 10^4 cm^{-3} and a cosmic ray ionization rate of 10^{-17} s^{-1} [e.g., 4]. Such low densities are unlikely at later evolutionary stages. For higher densities of 10^6 cm^{-3}, the cosmic ray ionization rate needs to exceed 10^{-15} s^{-1} to enhance the deuteration of water ice. As there are no indications for a cosmic ray ionization rate that is elevated by two orders of magnitude, an enhancement in the water deuteration in the protostellar envelope or protoplanetary disk phase of the V883 Ori system is not expected.

2.2 HDO/H_2O ratio

Using the reimaged HDO and $H_2^{18}O$ emission that was originally presented in [10], we derive the HDO/H_2O ratio to be $(3.7 \pm 1.0) \times 10^{-3}$. This is consistent within 1.2σ with that of $(2.3 \pm 0.6) \times 10^{-3}$ derived in [10] and their conclusions.

A summary of the observed HDO/H_2O ratios across Class 0, the reanalyzed datapoint in the V883 Ori disk, and comets is presented in Supplementary Data Figure 2 [7–26]. Recently it was found that the D/H ratio in water in the comet 67 P is lower than originally thought at a value of $(2.59 \pm 0.36) \times 10^{-4}$ (corresponding to HDO/H_2O = $(5.18 \pm 0.72) \times 10^{-4}$) due to the effect preferential adsorption of HDO on the dust grains [15]. As the D_2O abundance has not been rederived taking the effect of the dust into account, we use previously derived HDO/H_2O ratio of $(1.06 \pm 0.14) \times 10^{-3}$ for a consistent analysis across all water isotopologue ratios measured in this comet [11]. Using the newly derived HDO/H_2O ratio in the comet 67 P would increase the (D_2O/HDO)/(HDO/H_2O) ratio by a factor of 4 assuming that the D_2O does not preferentially adsorb on the dust grains like HDO. This does not change our conclusions. The color shading in the background of the left panel and the histogram on the right clearly demonstrate that the distributions of the expected HDO/H_2O ratio overlap for inheritance and reset and thus that this ratio does not distinguish inheritance from reset despite the similarity in the HDO/H_2O ratio across three evolutionary phases suggesting inheritance.

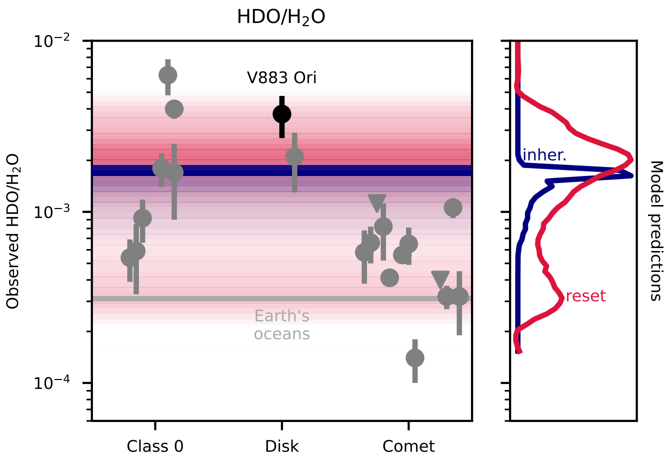

Supplementary Data Figure 2 The HDO/H_2O ratio across different stages of star and planet formation. The measurement in the V883 Ori disk are presented in black and those in the Class 0 objects NGC 1333 IRAS 2A, NGC 1333 IRAS 4A-NW, NGC 1333 IRAS 4B, IRAS 16293-2422, B335, BHR71-IRS1, L483, the L 1551 IRS5 disk, and the comets in grey [7–27]. The errorbars represent the 1σ uncertainty (s.d.) on the measured column density ratio in each source. The colored background and the histograms each normalized to the peak number of fluid parcels on the side indicate the expected water isotopologue ratios for inheritance where $\lesssim$ 10% of the H_2O ice is destroyed (blue) or reset where $\gtrsim$ 70% of the H_2O is expected to be destroyed through photodissociation and photodesorption in a model of a collapsing core (red; [1]). The red histogram is smoothed using a Savitzky-Golay filter with a window of 10 and an order of 3.

2.3 D_2O/HDO ratio

Another water isotopologue ratio is the D_2O/HDO ratio. This ratio in astronomical objects together with the model predictions by [1] are presented in Supplementary Data Figure 3. In case the material is inherited, the D_2O/HDO ratio is $\sim 2 \times 10^{-2}$, whereas in case the material is reset, the D_2O/HDO ratio is $\sim 10^{-4}$, though high values up $\sim 1 \times 10^{-2}$ are rare but still possible. The D_2O/HDO ratio in the V883 Ori disk is $(8.5 \pm 2.8) \times 10^{-3}$, much closer to the ratio expected for inheritance than for reset. In addition, this ratio is consistent within 1σ with that in the Class 0 sources L483 and NGC 1333 IRAS 2A, and with the comet 67 P and within 2σ of B335, the only other Class 0 source with an D_2O/HDO measurement. Therefore, the D_2O/HDO ratio in the V883 Ori disk is consistent with inheritance.

2.4 (D_2O/HDO) / (HDO/H_2O) ratio

Similarly, the overlap between the (D_2O/HDO) / (HDO/H_2O) ratio for inheritance and reset is much less than that for the HDO/H_2O ratio and therefore this is a better tracer than the HDO/H_2O ratio [1]. Model predictions show that the (D_2O/HDO) / (HDO/H_2O) ratio is $\sim 0.8 - 19$ if the material is inherited where the spread is due to e.g., variations in the initial ortho-to-para ratio of H_2, the abundances at the time at the time the prestellar core forms, and lifetime of the prestellar core before collapse [5]. In the collapsing core models by [1] the initial (D_2O/HDO) / (HDO/H_2O) ratio is

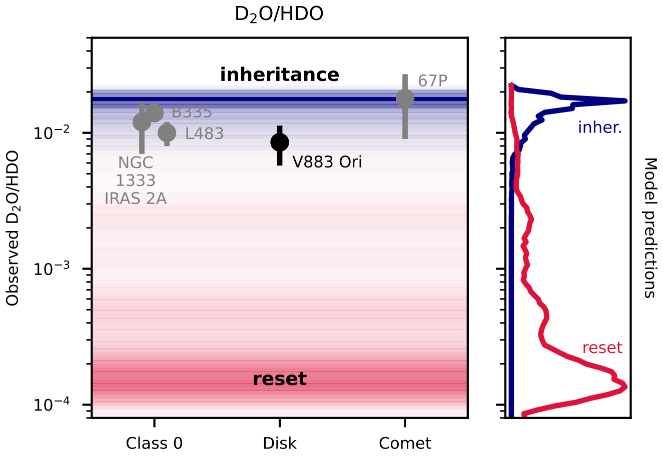

Supplementary Data Figure 3 The D_2O/HDO ratio across different stages of star and planet formation. The measurements in the V883 Ori disk are presented in black and those in the Class 0 objects NGC 1333 IRAS 2A, B335, L483, and the 67P comet in grey [7–9, 11, 12]. The errorbars represent the 1σ uncertainty (s.d.) on the measured column density ratio in each source. The colored background and the histograms each normalized to the peak number of fluid parcels on the side indicate the expected water isotopologue ratios for reset where $\gtrsim 70\%$ of the H_2O is expected to be destroyed through photodissociation and photodesorption in a model of a collapsing core (red; [1]). The red histogram is smoothed using a Savitzky-Golay filter with a window of 10 and an order of 3.

10. If material is reset, the expected value is $\sim 5 \times 10^{-2}$ and only few of the modeled stream lines predict a (D_2O/HDO) / (HDO/H_2O) above 1 in case of reprocessing (see Extended Data Figure 9) [1].

The observations of the (D_2O/HDO) / (HDO/H_2O) ratio in the V883 Ori disk together with those in younger Class 0 objects and the 67 P comet are presented in Extended Data Figure 9. All values are consistent with those for inheritance when variations in the initial conditions are considered [5]. Only NGC 1333 IRAS 2A and 67 P are consistent with the model prediction for inheritance by [1] within 1σ. The two remaining Class 0 sources and the V883 Ori disk have a somewhat lower value of $\sim 2 - 3$, possibly due to e.g., a different ortho-to-para ratio of H_2 and thus a different reservoir of chemical energy in the initial phases or due a different evolution of the early phases such as the time before the onset of collapse of the prestellar core and the visual extinction of the cloud when the initial conditions of the chemical network are initialized [5]. The similarity in the observed (D_2O/HDO) / (HDO/H_2O) ratio across Class 0 objects and the V883 Ori disk, together with the spread in possible values for inheritance strongly suggests that ice is preserved from the earliest phases of star and planet formation.

Supplementary Data Table 2 Effect of the excitation temperature on the D_2O/H_2O and the (D_2O/HDO) / (HDO/H_2O) ratios.

T_{rot} (K)	D_2O/H_2O	(D_2O/HDO) / (HDO/H_2O)
99	$(1.3 \pm 0.8) \times 10^{-5}$	1.4 ± 0.9
199	$(3.2 \pm 1.2) \times 10^{-5}$	2.3 ± 1.0
299	$(4.3 \pm 1.4) \times 10^{-5}$	2.7 ± 1.0

All uncertainties include the absolute flux calibration uncertainty of ALMA, the uncertainty on the assumed excitation temperature, and the uncertainty on the $^{16}O/^{18}O$ ratio.

3 Systematic uncertainties

In addition to the effect of the continuum optical depth potentially leading to an underestimation of the water isotopologue ratios discussed in the main text, other systematic effects could affect the derived ratios.

3.1 Excitation temperature

As only a single line of each water isotopologue is analyzed in this work, their measured column densities are derived using an assumed excitation temperature of 199 ± 42 K derived from a rotational diagram analysis of two HDO lines by [10]. As one HDO line at 241 GHz used in that analysis is severely blended with two COMs, the derived excitation temperature is somewhat uncertain. The temperature derived from these two HDO lines is higher than the typically assumed 120 K temperature for COMs in this disk based on their desorption temperature of ~ 100 K [28–31] and lower than the excitation temperature of 343 ± 102 K for CH_3CN, the warmest COM seen in this disk [29]. Supplementary Data Table 2 summarizes the effect of the assumed excitation temperature on the derived D_2O/H_2O and the (D_2O/HDO) / (HDO/H_2O) ratios by varying the temperature by ± 100 K, the approximate range of temperatures found for COMs in this disk. These ratios increase with temperature, driving the results further into inheritance if the gas is warmer than assumed. Even if the gas were only 99 K, the D_2O/H_2O ratio would be fully consistent with inheritance of material. In addition, the (D_2O/HDO) / (HDO/H_2O) ratio at this temperature is consistent within 1σ with those in Class 0 objects tracing pristine material.

3.2 Optical depth

The column densities of the water isotopologues are derived in the assumption of optically thin emission. The optical depth at the line center τ can be approximated as [32, 33]:

$$\tau = \frac{A_{ul} c^3}{8\pi\nu^3 \Delta V} N_u \left(e^{h\nu/kT_{\rm rot}} - 1 \right) \quad (1)$$

with ν the frequency of the line and ΔV the thermal linewidth. This expression approximates the peak of the normalized line profile as $1/\Delta V$ which is very close to that for a Gaussian line profile $2\sqrt{\ln 2}/(\sqrt{\pi}\Delta V)$. The thermal linewidth is defined as:

$$\Delta V = \sqrt{\frac{8kT_{\mathrm{rot}} \ln 2}{m}} \quad (2)$$

with m the mass of the molecule. For all three water isotopologues, this results in an FWHM linewidth of $\sim$ 0.7 km s^{-1} at a temperature of 199 K. This is slightly narrower than the line width in the shifted spectra because the finite resolution of the data and possible differences in the true emitting geometry set by the stellar mass, inclination, position angle, and assumed emitting height. An overview of the optical depth estimates is presented in Supplementary Data Table 3.

For the fiducial temperature of 199 K, the thermal linewidth and derived column densities of the upper energy levels, all lines analyzed in this work are optically thin. The HDO line has the highest optical depth at 0.14, which results in a 7% correction factor on the column density using the optical depth correction factor $\tau/(1 - e^{-\tau})$ [32, 33]. At a lower temperature of 99 K, the HDO line has an optical depth of 0.54 which leads to a correction factor of 29% on the HDO column density. This lowers the (D_2O/HDO) / (HDO/H_2O) ratio to 1.5 which is at the boundary of 1 between inheritance and reset. At a higher temperature of 299 K, all lines are fully optically thin. The best tracer to distinguish inheritance from reset used in this work, the D_2O/H_2O ratio, is not affected by the optical depth of HDO. Therefore, the observed D_2O and $H_2^{18}O$ emission remain consistent with the inheritance scenario even if the temperature is different from 199 ± 42 K.

Finally, the effect of the assumed emitting region for the emission is summarized in the final column of Supplementary Data Table 3. The fiducial region of $0''.4$ radius in the frame of the disk is motivated by the HDO and $H_2^{18}O$ emission that is seen out to that distance [10]. As the midplane water snowline is located at $\sim$80 au ($0''.2$) indicated by the steep drop off in the HDO and $H_2^{18}O$ column densities, the smallest physically motivated emitting region is an elliptical annulus between $0''.1$ and $0''.2$. The inner boundary is set by the optically thick dust that hides the line emission out to 40 au ($0''.1$).

The area of the annulus is a factor of 5.3 smaller than the fiducial region, increasing the derived column densities by that same factor. As all column densities change by that factor under the assumption of optically thin emission, the derived line ratios remain unaffected. The optical depths of the water isotopologue lines on the other hand increases by a factor of 5.3. For this small emitting region, the HDO emission becomes optically thick with an optical depth of 9.8×10^{-1}, making the water isotopologue ratios involving HDO hard to interpret as they are very sensitive to the precise optical depth and because the HDO possibly emits from a higher layer in the disk than the D_2O and $H_2^{18}O$. The D_2O and $H_2^{18}O$ on the other hand remain approximately optically thin with optical depths of 7.6×10^{-2} and 1.5×10^{-1}, respectively. Therefore, even in this case the D_2O/H_2O ratio can be used to infer that water is inherited.

Supplementary Data Table 3 Optical depth of the lines analyzed in this work.

molecule	τ $T_{\rm rot}$ = 199 K (fid.)	τ $T_{\rm rot}$ = 99 K	τ $T_{\rm rot}$ = 299 K	τ $T_{\rm rot}$ = 199 K & small emitting region
D_2O	1.4×10^{-2}	4.2×10^{-2}	7.6×10^{-3}	7.6×10^{-2}
HDO	1.8×10^{-1}	5.4×10^{-1}	9.9×10^{-2}	9.8×10^{-1}
$H_2^{18}O$	2.8×10^{-2}	8.0×10^{-2}	1.5×10^{-2}	1.5×10^{-1}

3.3 Ortho-to-para ratios

The D_2O and $H_2^{18}O$ molecules both have ortho and para states due to the presence of two deuterium and two hydrogen atoms, respectively. Observations of D_2O in the IRAS 16293-2422 cold protostellar envelope show that the observed value is consistent with the statistical value of 2 [34]. In addition, observations of the main water isotopologue show a similar trend with most observations being consistent with the statistical value of 3 [35, and references therein]. If the true ortho-to-para ratio (OPR) is different than the assumed statistical then the column density derived is scaled by a factor of $(1 + \mathrm{OPR})/3$ and $(1 + \mathrm{OPR})/4$, respectively. If the OPR is at a low value of 1 for both molecules, then the derived D_2O/H_2O increases to $(4.2 \pm 1.6) \times 10^{-5}$ and (D_2O/HDO) / (HDO/H_2O) ratio decreases to 0.8 ± 0.3. Even in this case, the D_2O/H_2O ratio remains consistent with inheritance.

3.4 Masing

The column densities of the water isotopologues are derived under the assumption of LTE. However, a recent analysis by [36] showed that the HDO 225 GHz and the $H_2^{18}O$ lines are possibly weakly masing in a large region in the V883 Ori disk. At the moment, detailed calculations of the masing conditions of the D_2O line are not reported. Still, the critical density for transitions of a non-linear, polyatomic molecule like the D_2O $1_{1,0} - 1_{0,1}$ transition at 200 K is 9.9×10^5 cm^{-3} using the collisional rate coefficients by [36]. This is much lower than the expected densities in the V883 Ori disk. Therefore, the D_2O $1_{1,0} - 1_{0,1}$ line is expected to be in LTE.

If the HDO and $H_2^{18}O$ lines are indeed weakly masing as suggested by [36], their column densities are overestimated due to the assumption of LTE for the rotational diagram analysis. In addition, the relation between column density and observed flux becomes highly sensitive to the local conditions. Therefore, the (D_2O/HDO) / (HDO/H_2O) is not a meaningful measure in this case. However, the D_2O/H_2O ratio can still be used as the potential masing of the $H_2^{18}O$ line only drives this ratio further into the inheritance regime whereas the D_2O line is expected to be in LTE.

References

[1] K. Furuya, M. N. Drozdovskaya, R. Visser, E. F. van Dishoeck, C. Walsh, D. Harsono, U. Hincelin, and V. Taquet. Water delivery from cores to disks: Deuteration as a probe of the prestellar inheritance of H_2O. *Astron. Astrophys.*, 599:A40, March 2017.

[2] William D. Watson. Interstellar molecule reactions. *Reviews of Modern Physics*, 48(4):513–552, October 1976.

[3] C. Ceccarelli, P. Caselli, D. Bockelée-Morvan, O. Mousis, S. Pizzarello, F. Robert, and D. Semenov. Deuterium Fractionation: The Ariadne's Thread from the Precollapse Phase to Meteorites and Comets Today. In *Protostars and Planets VI*, page 859, January 2014.

[4] Edwin A. Bergin and Mario Tafalla. Cold Dark Clouds: The Initial Conditions for Star Formation. *Annu. Rev. Astron. Astrophys.*, 45(1):339–396, September 2007.

[5] K. Furuya, E. F. van Dishoeck, and Y. Aikawa. Reconstructing the history of water ice formation from HDO/H_2O and D_2O/HDO ratios in protostellar cores. *Astron. Astrophys.*, 586:A127, February 2016.

[6] S. D. Rodgers and S. B. Charnley. Multiply deuterated molecules and constraints on interstellar chemistry. *Planet. Space Sci.*, 50(12-13):1125–1132, October 2002.

[7] S. S. Jensen, J. K. Jørgensen, L. E. Kristensen, K. Furuya, A. Coutens, E. F. van Dishoeck, D. Harsono, and M. V. Persson. ALMA observations of water deuteration: a physical diagnostic of the formation of protostars. *Astron. Astrophys.*, 631:A25, November 2019.

[8] S. S. Jensen, J. K. Jørgensen, L. E. Kristensen, A. Coutens, E. F. van Dishoeck, K. Furuya, D. Harsono, and M. V. Persson. ALMA observations of doubly deuterated water: inheritance of water from the prestellar environment. *Astron. Astrophys.*, 650:A172, June 2021.

[9] A. Coutens, J. K. Jørgensen, M. V. Persson, E. F. van Dishoeck, C. Vastel, and V. Taquet. High D_2O/HDO Ratio in the Inner Regions of the Low-mass Protostar NGC 1333 IRAS2A. *Astrophys. J. Lett.*, 792(1):L5, September 2014.

[10] John J. Tobin, Merel L. R. van't Hoff, Margot Leemker, Ewine F. van Dishoeck, Teresa Paneque-Carreño, Kenji Furuya, Daniel Harsono, Magnus V. Persson, L. Ilsedore Cleeves, Patrick D. Sheehan, and Lucas Cieza. Deuterium-enriched water ties planet-forming disks to comets and protostars. *Nature*, 615(7951):227–230, March 2023.

[11] K. Altwegg, H. Balsiger, J. J. Berthelier, A. Bieler, U. Calmonte, J. De Keyser, B. Fiethe, S. A. Fuselier, S. Gasc, T. I. Gombosi, T. Owen, L. Le Roy, M. Rubin, T. Sémon, and C. Y. Tzou. D_2O and HDS in the coma of 67P/Churyumov-Gerasimenko. *RSPTA*, 375(2097):20160253, May 2017.

[12] K. Altwegg, H. Balsiger, A. Bar-Nun, J. J. Berthelier, A. Bieler, P. Bochsler, C. Briois, U. Calmonte, M. Combi, J. De Keyser, P. Eberhardt, B. Fiethe, S. Fuselier, S. Gasc, T. I. Gombosi, K. C. Hansen, M. Hässig, A. Jäckel, E. Kopp, A. Korth, L. LeRoy, U. Mall, B. Marty, O. Mousis, E. Neefs,

T. Owen, H. Rème, M. Rubin, T. Sémon, C. Y. Tzou, H. Waite, and P. Wurz. 67P/Churyumov-Gerasimenko, a Jupiter family comet with a high D/H ratio. *Science*, 347(6220):1261952, January 2015.

[13] R. Hagemann, G. Nief, and E. Roth. Absolute isotopic scale for deuterium analysis of natural waters. Absolute D/H ratio for SMOW. *Tellus*, 22(6):712–715, December 1970.

[14] M. V. Persson, J. K. Jørgensen, E. F. van Dishoeck, and D. Harsono. The deuterium fractionation of water on solar-system scales in deeply-embedded low-mass protostars. *Astron. Astrophys.*, 563:A74, March 2014.

[15] Kathleen E. Mandt, Jacob Lustig-Yaeger, Adrienn Luspay-Kuti, Peter Wurz, Dennis Bodewits, Stephen A. Fuselier, Olivier Mousis, Steven M. Petrinec, and Karlheinz J. Trattner. A nearly terrestrial d/h for comet 67p/churyumov-gerasimenko. *Science Advances*, 10(46):eadp2191, 2024.

[16] John R. de Laeter, John Karl Böhlke, P. De Bièvre, H. Hidaka, H. S. Peiser, K. J. R. Rosman, and P. D. P. Taylor. Atomic weights of the elements. review 2000 (iupac technical report). *Pure and Applied Chemistry*, 75(6):683–800, 2003.

[17] D. Bockelée-Morvan, D. Gautier, D. C. Lis, K. Young, J. Keene, T. Phillips, T. Owen, J. Crovisier, P. F. Goldsmith, E. A. Bergin, D. Despois, and A. Wootten. Deuterated Water in Comet C/1996 B2 (Hyakutake) and Its Implications for the Origin of Comets. *Icarus*, 133(1):147–162, May 1998.

[18] Roland Meier, Tobias C. Owen, Henry E. Matthews, David C. Jewitt, Dominique Bockelee-Morvan, Nicolas Biver, Jacques Crovisier, and Daniel Gautier. A Determination of the HDO/H2O Ratio in Comet C/1995 O1 (Hale-Bopp). *Science*, 279:842, February 1998.

[19] Erika L. Gibb, Boncho P. Bonev, Geronimo Villanueva, Michael A. DiSanti, Michael J. Mumma, Emily Sudholt, and Yana Radeva. Chemical Composition of Comet C/2007 N3 (Lulin): Another "Atypical" Comet. *Astrophys. J.*, 750(2):102, May 2012.

[20] G. L. Villanueva, M. J. Mumma, B. P. Bonev, M. A. Di Santi, E. L. Gibb, H. Böhnhardt, and M. Lippi. A Sensitive Search for Deuterated Water in Comet 8p/Tuttle. *Astrophys. J. Lett.*, 690(1):L5–L9, January 2009.

[21] D. Bockelée-Morvan, N. Biver, B. Swinyard, M. de Val-Borro, J. Crovisier, P. Hartogh, D. C. Lis, R. Moreno, S. Szutowicz, E. Lellouch, M. Emprechtinger, G. A. Blake, R. Courtin, C. Jarchow, M. Kidger, M. Küppers, M. Rengel, G. R. Davis, T. Fulton, D. Naylor, S. Sidher, and H. Walker. Herschel measurements of the D/H and $^{16}O/^{18}O$ ratios in water in the Oort-cloud comet C/2009 P1 (Garradd). *Astron. Astrophys.*, 544:L15, August 2012.

[22] N. Biver, D. Bockelée-Morvan, J. Crovisier, D. C. Lis, R. Moreno, P. Colom, F. Henry, F. Herpin, G. Paubert, and M. Womack. Radio wavelength molecular observations of comets C/1999 T1 (McNaught-Hartley), C/2001 A2 (LINEAR), C/2000 WM_1 (LINEAR) and 153P/Ikeya-Zhang. *Astron. Astrophys.*, 449(3):1255–1270, April 2006.

[23] N. Biver, R. Moreno, D. Bockelée-Morvan, Aa. Sandqvist, P. Colom, J. Crovisier, D. C. Lis, J. Boissier, V. Debout, G. Paubert, S. Milam, A. Hjalmarson, S. Lundin, T. Karlsson, M. Battelino, U. Frisk, D. Murtagh, and Odin Team. Isotopic ratios of H, C, N, O, and S in comets C/2012 F6 (Lemmon) and C/2014 Q2 (Lovejoy). *Astron. Astrophys.*, 589:A78, May 2016.

[24] D. C. Lis, N. Biver, D. Bockelée-Morvan, P. Hartogh, E. A. Bergin, G. A. Blake, J. Crovisier, M. de Val-Borro, E. Jehin, M. Küppers, J. Manfroid, R. Moreno, M. Rengel, and S. Szutowicz. A Herschel Study of D/H in Water in the Jupiter-family Comet 45P/Honda-Mrkos-Pajdušáková and Prospects for D/H Measurements with CCAT. *Astrophys. J. Lett.*, 774(1):L3, September 2013.

[25] Paul Hartogh, Dariusz C. Lis, Dominique Bockelée-Morvan, Miguel de Val-Borro, Nicolas Biver, Michael Küppers, Martin Emprechtinger, Edwin A. Bergin, Jacques Crovisier, Miriam Rengel, Raphael Moreno, Slawomira Szutowicz, and Geoffrey A. Blake. Ocean-like water in the Jupiter-family comet 103P/Hartley 2. *Nature*, 478(7368):218–220, October 2011.

[26] Dariusz C. Lis, Dominique Bockelée-Morvan, Rolf Güsten, Nicolas Biver, Jürgen Stutzki, Yan Delorme, Carlos Durán, Helmut Wiesemeyer, and Yoko Okada. Terrestrial deuterium-to-hydrogen ratio in water in hyperactive comets. *Astron. Astrophys.*, 625:L5, May 2019.

[27] A. Andreu, A. Coutens, F. Cruz-Sáenz de Miera, N. Houry, J. K. Jørgensen, A. Kóspál, and D. Harsono. A high HDO/H_2O ratio in the Class I protostar L1551 IRS5. *Astron. Astrophys.*, 677:L17, September 2023.

[28] Jeong-Eun Lee, Seokho Lee, Giseon Baek, Yuri Aikawa, Lucas Cieza, Sung-Yong Yoon, Gregory Herczeg, Doug Johnstone, and Simon Casassus. The ice composition in the disk around V883 Ori revealed by its stellar outburst. *Nature Astronomy*, 3:314–319, February 2019.

[29] Jae-Hong Jeong, Jeong-Eun Lee, Seonjae Lee, Giseon Baek, Ji-Hyun Kang, Seokho Lee, Chul-Hwan Kim, Hyeong-Sik Yun, Yuri Aikawa, Gregory J. Herczeg, Doug Johnstone, and Lucas Cieza. ALMA Spectral Survey of an Eruptive Young Star, V883 Ori (ASSAY). II. Freshly Sublimated Complex Organic Molecules in the Keplerian Disk. *Astrophys. J. Suppl. Ser.*, 276(2):49, February 2025.

[30] Yoshihide Yamato, Shota Notsu, Yuri Aikawa, Yuki Okoda, Hideko Nomura, and Nami Sakai. Chemistry of Complex Organic Molecules in the V883 Ori Disk Revealed by ALMA Band 3 Observations. *Astron. J.*, 167(2):66, February 2024.

[31] Merel L. R. van 't Hoff, John J. Tobin, Leon Trapman, Daniel Harsono, Patrick D. Sheehan, William J. Fischer, S. Thomas Megeath, and Ewine F. van Dishoeck. Methanol and its Relation to the Water Snowline in the Disk around the Young Outbursting Star V883 Ori. *Astrophys. J. Lett.*, 864(1):L23, September 2018.

[32] Paul F. Goldsmith and William D. Langer. Population Diagram Analysis of Molecular Line Emission. *Astrophys. J.*, 517(1):209–225, May 1999.

[33] Ryan A. Loomis, L. Ilsedore Cleeves, Karin I. Öberg, Yuri Aikawa, Jennifer Bergner, Kenji Furuya, V. V. Guzman, and Catherine Walsh. The Distribution and Excitation of CH_3CN in a Solar Nebula Analog. *Astrophys. J.*, 859(2):131, June 2018.

[34] C. Vastel, C. Ceccarelli, E. Caux, A. Coutens, J. Cernicharo, S. Bottinelli, K. Demyk, A. Faure, L. Wiesenfeld, Y. Scribano, A. Bacmann, P. Hily-Blant, S. Maret, A. Walters, E. A. Bergin, G. A. Blake, A. Castets, N. Crimier, C. Dominik, P. Encrenaz, M. Gérin, P. Hennebelle, C. Kahane, A. Klotz, G. Melnick, L. Pagani, B. Parise, P. Schilke, V. Wakelam, A. Baudry, T. Bell, M. Benedettini, A. Boogert, S. Cabrit, P. Caselli, C. Codella, C. Comito, E. Falgarone, A. Fuente, P. F. Goldsmith, F. Helmich, T. Henning, E. Herbst, T. Jacq, M. Kama, W. Langer, B. Lefloch, D. Lis, S. Lord, A. Lorenzani, D. Neufeld, B. Nisini, S. Pacheco, J. Pearson, T. Phillips, M. Salez, P. Saraceno, K. Schuster, X. Tielens, F. van der Tak, M. H. D. van der Wiel, S. Viti, F. Wyrowski, H. Yorke, P. Cais, J. M. Krieg, M. Olberg, and L. Ravera. Ortho-to-para ratio of interstellar heavy water. *Astron. Astrophys.*, 521:L31, October 2010.

[35] A. Faure, P. Hily-Blant, C. Rist, G. Pineau des Forêts, A. Matthews, and D. R. Flower. The ortho-to-para ratio of water in interstellar clouds. *Mon. Not. R. Astron. Soc.*, 487(3):3392–3403, August 2019.

[36] A. Faure, M. Żółtowski, L. Wiesenfeld, F. Lique, and A. Bergeat. The rotational excitation of the water isotopologues by molecular hydrogen. *Mon. Not. R. Astron. Soc.*, 527(2):3087–3093, January 2024.
